# Trachoma elimination in Latin America: prioritization of municipalities for surveillance activities

**DOI:** 10.26633/RPSP.2019.93

**Published:** 2019-12-12

**Authors:** Martha Idalí Saboyá-Díaz, Angel F Betanzos-Reyes, Sheila K West, Beatriz Muñoz, Luis Gerardo Castellanos, Marcos Espinal

**Affiliations:** 1 Communicable Diseases and Environmental Determinants of Health Department Pan American Health Organization/World Health Organization Washington, DC United States of the America Communicable Diseases and Environmental Determinants of Health Department, Pan American Health Organization/World Health Organization, Washington, DC, United States of the America.; 2 Centro de Investigación sobre Enfermedades Infecciosas Instituto Nacional de Salud Pública de México CuernavacaMorelos Mexico Centro de Investigación sobre Enfermedades Infecciosas, Instituto Nacional de Salud Pública de México, Cuernavaca, Morelos, Mexico.; 3 Dana Center for Preventive Ophthalmology Wilmer Eye Institute, Johns Hopkins Hospital BaltimoreMaryland United States of America Dana Center for Preventive Ophthalmology, Wilmer Eye Institute, Johns Hopkins Hospital, Baltimore, Maryland, United States of America.

**Keywords:** Trachoma, neglected diseases, surveillance, Latin America, Tracoma, enfermedades desatendidas, vigilancia, América Latina, Tracoma, doenças negligenciadas, vigilância, América Latina

## Abstract

**Objective.:**

To identify and prioritize municipalities in 22 countries of Latin America for trachoma surveillance activities, to measure the absence or prevalence of trachoma, and to support validation and trachoma elimination efforts in the Region of the Americas.

**Methods.:**

A prioritization scale was developed in 2017 to rank each municipality by considering a combination of three characteristics: (a) its trachoma vulnerability index, derived from three socioeconomic factors known to be risks for trachoma—lack of access to improved sanitation, to clean drinking water, and to adequate education, according to housing census data from early 2017; (b) its history of trachoma in countries where the disease was not a known public health problem in 2016; and (c) whether or not it shares a border with a municipality where trachoma was a known public health problem in 2016. Municipalities in 22 countries were classified as either very high, high, medium, or low priority for trachoma surveillance. From the Caribbean, only Trinidad and Tobago met inclusion criteria.

**Results.:**

The prioritization scale identified 1 053 municipalities in Brazil, Colombia, and Guatemala as *very high* priority for trachoma surveillance. In Ecuador, El Salvador, Guyana, Paraguay, Peru, Suriname, and Venezuela, 183 municipalities were ranked as *high* priority, and in Argentina, Belize, Bolivia, Chile, Dominican Republic, Honduras, Nicaragua, Panama, and Uruguay, 677 municipalities were designated a *medium* priority for trachoma surveillance.

**Conclusions.:**

This prioritization scale will be useful to countries in Latin America that still need to ascertain their current trachoma situation. The absence or prevalence of trachoma in countries designated as very high and high priority for trachoma surveillance activities must be studied to determine the extent of the disease in Latin America.

## RESUMEN

**Objetivo.:**

Establecer y priorizar los municipios en 22 países de América Latina para desempeñar actividades de vigilancia del tracoma, medir la ausencia o la prevalencia del tracoma, y brindar apoyo a la validación y las iniciativas de eliminación del tracoma en la Región de las Américas.

**Métodos.:**

En el 2017, se creó una escala de asignación de prioridades con el fin de clasificar cada municipio teniendo presente una combinación de tres características: *a*) el índice de vulnerabilidad al tracoma, derivado de los tres factores socioeconómicos conocidos por ser factores de riesgos para el tracoma (falta de acceso del municipio a mejores instalaciones sanitarias, agua potable limpia y educación adecuada), según los datos del censo de vivienda de principios del 2017; *b*) los antecedentes de tracoma en los países en que la enfermedad no se conocía como problema de salud pública en el año 2016, y *c*) si el municipio comparte frontera con otro donde el tracoma supuso un problema de salud pública en el 2016. Se clasificaron municipios de 22 países según un criterio de prioridad muy alta, alta, media o baja de vigilancia del tracoma. En el Caribe, únicamente Trinidad y Tabago cumplieron los criterios de inclusión.

**Resultados.:**

Mediante la escala de asignación de prioridades se establecieron 1 053 municipios en Brasil, Colombia y Guatemala con prioridad *muy alta* de vigilancia del tracoma. En Ecuador, El Salvador, Guyana, Paraguay, Perú, Suriname y Venezuela, 183 municipios se clasificaron con prioridad *alta* y en Argentina, Belice, Bolivia, Chile, República Dominicana, Honduras, Nicaragua, Panamá y Uruguay, se asignó a 677 municipios una prioridad *media* de vigilancia del tracoma.

**Conclusiones.:**

Esta escala de asignación de prioridades será una herramienta útil para los países en América Latina que todavía necesitan evaluar su actual situación de tracoma. Debe estudiarse la ausencia o prevalencia del tracoma en los países designados con prioridades muy alta y alta para las actividades de vigilancia de tracoma con el fin de determinar la magnitud de la enfermedad en América Latina.

## RESUMO

**Objetivo.:**

Identificar e priorizar os municípios em 22 países da América Latina para realizar atividades de vigilância epidemiológica do tracoma, determinar a ausência ou a prevalência do tracoma e dar apoio à validação e aos esforços de eliminação da doença na Região das Américas.

**Métodos.:**

Uma escala de priorização foi elaborada em 2017 para classificar cada município segundo três critérios combinados: (a) índice de vulnerabilidade ao tracoma, estimado com base em três fatores socioeconômicos conhecidos como riscos à doença – falta de acesso a um melhor saneamento básico, à água para consumo e a uma educação adequada, segundo dados do censo habitacional realizado no início de 2017; (b) antecedentes do tracoma nos países onde a doença não era um problema conhecido de saúde pública em 2016 e (c) fazer divisa ou não com um município onde o tracoma era um problema conhecido de saúde pública em 2016. Os municípios de 22 países foram classificados como de prioridade muito alta, alta, média ou baixa para a vigilância do tracoma. No Caribe, apenas Trinidad e Tobago satisfez os critérios de inclusão.

**Resultados.:**

De acordo com a escala de priorização, 1.053 municípios no Brasil, Colômbia e Guatemala foram classificados como de prioridade *muito alta*; 183 municípios no Equador, El Salvador, Guiana, Paraguai, Peru, Suriname e Venezuela como de prioridade *alta*, e 677 municípios na Argentina, Belize, Bolívia, Chile, República Dominicana, Honduras, Nicarágua, Panamá e Uruguai foram qualificados como de prioridade *média* para a vigilância do tracoma.

**Conclusões.:**

A escala de priorização será um instrumento útil aos países na América Latina que ainda precisam determinar sua situação atual em relação ao tracoma. A ausência ou a prevalência da doença deve ser pesquisada nos países classificados como de prioridade muito alta e alta para as atividades de vigilância para que se possa conhecer a extensão do tracoma na América Latina.

Trachoma is a neglected tropical disease and the world’s leading infectious cause of blindness. Caused by certain serotypes of the *Chlamydia trachomatis* bacterium, it primarily affects populations that live in poverty, without access to safe water and basic sanitation (1). Active trachoma, characterized by the presence of subepithelial follicles—trachomatous inflammation-follicular (TF) and/or trachomatous inflammation-intense (TI)—is usually found in children. Repeated infections can scar the eyelids, which in some individuals causes the eyelashes from the upper eyelid to touch the eye (trachomatous trichiasis [TT]), eventually leading to corneal opacity and blindness (2). Given that trachoma is a public health problem in 44 countries and trichiasis affects about 2.5 million people, a global goal was set to eliminate trachoma as a public health problem by 2020 (3).

In Latin America, trachoma is known to be a public health problem in certain parts of four countries: Brazil, Colombia, Guatemala, and Peru; trachoma elimination was validated in Mexico in January 2017 (3). However, there are population groups living in vulnerable conditions in other countries of the Region and in other parts of Brazil, Colombia, Guatemala, and Peru. This situation indicates a need to measure the extent of the trachoma problem.

The objective of this study was to identify and prioritize municipalities in 22 countries of Latin America for trachoma surveillance activities, to measure the absence or prevalence of trachoma, and to support validation and trachoma elimination efforts in the Region of the Americas.

## MATERIALS AND METHODS

### Study design

This was an observational, retrospective study carried out in 2017 to develop a prioritization scale to rank municipalities of 22 countries of Latin America for trachoma surveillance. The study considered and combined: data on trachoma risk factors (lack of access to improved sanitation facilities, to improved drinking water, and to adequate education) from housing censuses; historical data on the disease published in 1987 – 2015 for countries where trachoma was not a known public health problem in 2016; and trachoma data from countries where it was a public health problem in 2016 (Brazil, Colombia, and Guatemala), as reported to the Pan American Health Organization/World Health Organization (PAHO/WHO). These data would likely locate all municipalities known to have trachoma and their bordering municipalities, both within and beyond each country.

Mexico was excluded from the prioritization scale because trachoma elimination was achieved in 2016 and validated in 2017 by PAHO/WHO. Peru was included as a country “not known to have trachoma as a public health problem in 2016,” according to its first baseline survey in 2017.

### Prioritization scale

A prioritization scale was constructed to identify municipalities in 22 countries of Latin America that need to carry out trachoma surveillance activities. The prioritization scale considered three characteristics of each municipality: (a) a trachoma vulnerability index derived from three socioeconomic factors known to put individuals at risk for trachoma (4); (b) the history of trachoma in countries where it was not a known public health problem in 2016 (hereafter “countries not known to have trachoma”); and (c) whether or not the municipality borders a municipality where trachoma is a known public health problem. Countries not known to have trachoma were those without evidence of TF prevalence ≥ 5% in children 1 – 9 years of age (estimated through population-based surveys) in any municipality in 2016. A municipality known to have trachoma was one with TF prevalence ≥ 5% in children 1 – 9 years of age or TT prevalence “unknown to the health system” ≥ 0.2% among those 15 years of age or older (estimated through population-based surveys) (5). Finally, municipalities were classified by levels of priority for trachoma surveillance.

The following describes the methodology in greater detail:

#### Trachoma Vulnerability Index (TVI).

The TVI was constructed on the basis of three selected risk factors: lack of access to (a) improved sanitation facilities, (b) improved drinking water, and (c) adequate education. In the first quarter of 2017, the available sources of data on the three factors were reviewed for municipalities (provinces, districts, municipalities, cantons, etc., depending on each country’s nomenclature and structure) in Latin America. Population and housing censuses were reviewed in 22 countries. In 17 countries, publicly available census databases were consulted (6 – 22). In the other five countries (Belize, Guatemala, Guyana, Nicaragua, and Suriname), databases compiled by a previous study were used (23). From the Caribbean, only Trinidad and Tobago was included; the other countries were excluded due to a lack of publicly available data on the three risk factors at the municipality level.

Data were compiled at the municipality level, and included all urban and rural areas:

- Number and proportion of households with unimproved sanitation facilities, including pit latrines without a slab/platform and hanging or bucket latrines. Data were available for 10 027 municipalities in 21 countries (missing data for all of Suriname and one municipality in Argentina).
- Number and proportion of households with unimproved drinking water sources, including those with an unprotected dug well, unprotected spring, cart with small tank/drum, tanker truck, and bottled water. Data were available for 10 089 municipalities in 22 countries (missing data for one municipality in Argentina).
- Number and proportion of individuals with inadequate education, defined as being more than 15 years of age and unable to read and write (no missing data).

The mean of the risk factor proportions was calculated for each municipality to produce its TVI. Then, the mean of all the TVIs for all municipalities was calculated, as well as the distribution by percentiles. The 50th percentile was chosen as the cut-off for TVI grouping categories. TVI values below the 50th percentile were considered low; from the 50th to less than the 75th were considered moderate; and 75^th^ and greater were considered high.

#### Historical reporting in countries not known to have trachoma.

Historical information on trachoma occurrence in Latin American countries not known to have had the disease in 2016 (not Brazil, Colombia, Guatemala, and Mexico) was compiled. In all, 200 articles met the criteria for a review of literature, which was conducted by the Dana Center for Preventive Ophthalmology, Wilmer Eye Institute, Johns Hopkins Hospital (data not yet published). Table 1 details criteria for the literature review (with permission from the authors).

**TABLE 1.** Criteria for a review of literature on trachoma in the Americas conducted by the Dana Center for Preventive Ophthalmology, 2015

Criteria	Details
Databases for electronic search	MEDLINE, Embase, LILACS, SCOPUS, and Web of Science; PAHO/WHO library databases; and unpublished gray literature from websites of Ministries of Health, tropical/infectious diseases institutions, and biomedical institutions.
Language restrictions	None.
Date restrictions	Scientific literature published until 13 July 2015.
Search terms	For MEDLINE, EMBASE, SCOPUS, LILACS and Web of Science: trachoma OR scarring trachoma OR entropion OR trichiasis OR ocular chlamydia OR corneal opacity OR blindness survey AND name of the country. For MEDLINE, exclusion words added for the United States were NOT sexual NOT Sexually transmitted disease NOT *Neisseria gonorrhoeae* NOT animal. For EMBASE, search was restricted to humans. For LILACS, search term Trachoma was added.
Inclusion criteria	(a) Population studied: school children, clusters within populations, and state/province or national surveys. (b) Type of study: historical data (pre-1940), single case report, clinical case series, risk factors study, blindness survey, trachoma prevalence survey, and clinical trials. (c) Outcomes: cases of trachoma, based on the WHO trachoma grading system or the Macallan/modified-Macallan grading system, or “unknown” if the outcomes were measured prior to 1930; and/or prevalence of trachoma.

***Source:*** Prepared by the authors with permission from the authors of the (unpublished) original study.

For the purpose of our study, a subset of articles was selected based on the following criteria:

- Time frame: studies conducted in 1987 – 2015. This time period was chosen primarily because the trachoma grading scale currently recommended by WHO was standardized in 1987 (2).
- Participants: persons who have been evaluated clinically or serologically for trachoma in any age group.
- Outcomes: cases of trachoma based on the WHO trachoma grading system (2), or prevalence of trachoma, or the detection of *C. trachomatis* antibodies.
- Type of study: any. Two reviewers (MIS and AFB) selected the studies independently and resolved disagreements by discussion and consensus. Full-text articles were analyzed. Nine articles met the inclusion criteria (Table 2).

**TABLE 2.** Information of the 9 studies included in the review of the historical occurrence of trachoma in Latin Americas, 1987 – 2015

Country	Author	Year of Publication	Study Type	Setting	Population	Outcome Measured	Geographical level of data reported		
							National	First administrative level	Second administrative level
Argentina	Barrenechea et al.	2015	Blindness survey	National population-based study. Clusters	Adults ≥50 years old	Prevalence of blindness causes	Argentina	—	—
El Salvador	Rius et al	2014	Blindness survey	Population-based survey RAAB methodology	Adults ≥50 years old	Prevalence of blindness causes	El Salvador	—	—
Haiti	Goodhew	2012	Cross sectional/ cohort study	Villages outside Leogane	Children <5 years old	Seroprevalence of CT	Haiti	—	—
Honduras	Alvarado et al.	2014	Blindness survey	Population-based survey RAAB methodology	Adults ≥50 years old	Prevalence of blindness causes	Honduras	—	—
Paraguay	Duerksen et al	2013	Blindness survey	Population-based survey RAAB methodology	Adults ≥50 years old	Prevalence of blindness causes	Paraguay	—	—
Peru	Munoz et al	2007	Case report	Patients from the National Ophthalmological Institute, Lima	Adults 18-68 years old of indigenous population (Shipiba)	Frequency of infection by CT (Giemsa, direct immunofluorescence, culture)	Peru	—	—
Peru	Campos et al	2014	Blindness survey	Population-based survey RAAB methodology	Adults ≥50 years old	Prevalence of blindness causes	Peru	—	—
Peru	Maco et al	2016	Literature Review	History of Trachoma in Peru	Indigenous and non-indigenous population	Any type of trachoma	Peru	—	—
Peru	Maco et al	2016	Cross sectional	Population-based survey	Unspecified	Any type of trachoma	Peru	Ucayali	San Francisco de Yarinacocha
Peru	Maco et al	2016	Cross sectional	Population-based survey	Unspecified	Any type of trachoma	Peru	Ucayali	Santa Teresita
Peru	Maco et al	2016	Case series	Unspecified	Unspecified	Any type of trachoma	Peru	Ucayali	Misión de Cashivococha
Peru	Maco et al	2016	Case series	Unspecified	Unspecified	Any type of trachoma	Peru	Loreto	Manati II
Peru	Maco et al	2016	Case series	Hospital, population-based surveys, outpatient clinic	Unspecified	Any type of trachoma	Peru	Lima	Comas
Peru	Maco et al	2016	Case series	Hospital, population-based surveys, outpatient clinic	Unspecified	Any type of trachoma	Peru	Lima	Canto Grande
Peru	Maco et al	2016	Case series	Hospital, population-based surveys, outpatient clinic	Unspecified	Any type of trachoma	Peru	Lima	Independencia
Peru	Maco et al	2016	Case series	Hospital, population-based surveys, outpatient clinic	Unspecified	Any type of trachoma	Peru	Lima	San Martin de Porras
Peru	Maco et al	2016	Case series	Hospital, population-based surveys, outpatient clinic	Unspecified	Any type of trachoma	Peru	Lima	San Luis
Peru	Maco et al	2016	Case series	Hospital, population-based surveys, outpatient clinic	Unspecified	Any type of trachoma	Peru	Lima	Pueblo Libre
Peru	Maco et al	2016	Case series	Hospital, population-based surveys, outpatient clinic	Unspecified	Any type of trachoma	Peru	Lima	La Molina
Peru	Maco et al	2016	Case series	Hospital, population-based surveys, outpatient clinic	Unspecified	Any type of trachoma	Peru	La Libertad	San Pedro de Lloc
Peru	Maco et al	2016	Case series	Hospital, population-based surveys, outpatient clinic	Unspecified	Any type of trachoma	Peru	Loreto	Iquitos
Peru	Maco et al	2016	Case series	Hospital, population-based surveys, outpatient clinic	Unspecified	Any type of trachoma	Peru	Junin	La Merced
Peru	Maco et al	2016	Case series	Hospital, population-based surveys, outpatient clinic	Unspecified	Any type of trachoma	Peru	Junin	San Ramon
Peru	Maco et al	2016	Case series	Hospital, population-based surveys, outpatient clinic	Unspecified	Any type of trachoma	Peru	Ucayali	Pucallpa
Peru	Maco et al	2016	Case series	Hospital, population-based surveys, outpatient clinic	Unspecified	Any type of trachoma	Peru	Ayacucho	Mala
Peru	Maco et al	2016	Retrospective	Population survey, outpatient clinic	Unspecified	Any type of trachoma	Peru	Lima	—
Peru	Maco et al	2016	Retrospective	Outpatient clinic	Unspecified	Any type of trachoma	Peru	Ancash	Huaraz
Peru	Maco et al	2016	Retrospective	Population-based survey	Unspecified	Any type of trachoma	Peru	Several departments	—
Peru	Maco et al	2016	Retrospective	Population-based survey	Unspecified	Any type of trachoma	Peru	Lima	—
Peru	Maco et al	2016	Retrospective	Population-based survey	Unspecified	Any type of trachoma	Peru	Ica	—
Peru	Maco et al	2016	Retrospective	Population-based survey	Unspecified	Any type of trachoma	Peru	Junin	—
Peru	Maco et al	2016	Retrospective	Population-based survey	Unspecified	Any type of trachoma	Peru	Huanuco	—
Peru	Maco et al	2016	Retrospective	Population-based survey	Unspecified	Any type of trachoma	Peru	San Martin	—
Peru	Maco et al	2016	Cross sectional	Population-based survey	Unspecified	Any type of trachoma	Peru	Ucayali	Calleria
Uruguay	Gallarreta et al	2014	Blindness survey	Population-based survey RAAB methodology	Adults ≥50 years old	Prevalence of blindness causes	Uruguay	—	—

CT: *Chlamydia trachomatis*

RAAB: Rapid assessment of avoidable blindness

(-): no data reported at this level

***Source:*** Prepared by the authors from the study results.

If an article reported data disaggregated from multiple geographic places, it provided more than one observation for the analysis; but, if the article reported data from a given country without identifying where trachoma had occurred, it counted as only one observation and the data was assumed to be applicable to each municipality in the country.

#### Municipalities bordering municipalities known to have trachoma.

The trachoma data that countries reported to PAHO/WHO in 2016 provided the names of the municipalities known to have trachoma in Brazil, Colombia, and Guatemala. Then, using the PAHO/WHO atlas of neglected infectious diseases (24), the municipalities known to have trachoma were located, as were the bordering municipalities within and outside the country.

#### Prioritization scale for municipalities requiring trachoma surveillance activities.

The 22 countries were divided into two groups: countries that WHO classified (25) as needing trachoma interventions in 2016 (Brazil, Colombia, and Guatemala), i.e., known to have trachoma; and the rest of the countries, i.e., not known to have trachoma.

In the countries known to have trachoma, the municipality prioritization scale was constructed by combining the TVI category (high, moderate, and low) with whether or not the municipality bordered a municipality/ies known to have trachoma. The prioritization scale resulted in four levels: very high, high, medium, and low. Although the combination of criteria for the prioritization scale was done arbitrarily, more importance was given to sharing a border with a municipality known to have trachoma. Many such municipalities in Brazil, Colombia, and Guatemala have semi-nomadic indigenous populations with high migration flows between municipalities (26).

Countries not known to have trachoma were separated into two subgroups: countries with historical data on the presence of trachoma published in 1987 – 2015, and countries without any data published in that period. For these two subgroups, the municipality prioritization scale was constructed by combining the TVI categories with whether or not a municipality bordered a municipality in a country known to have trachoma (Brazil, Colombia, and Guatemala).

**FIGURE 1.**
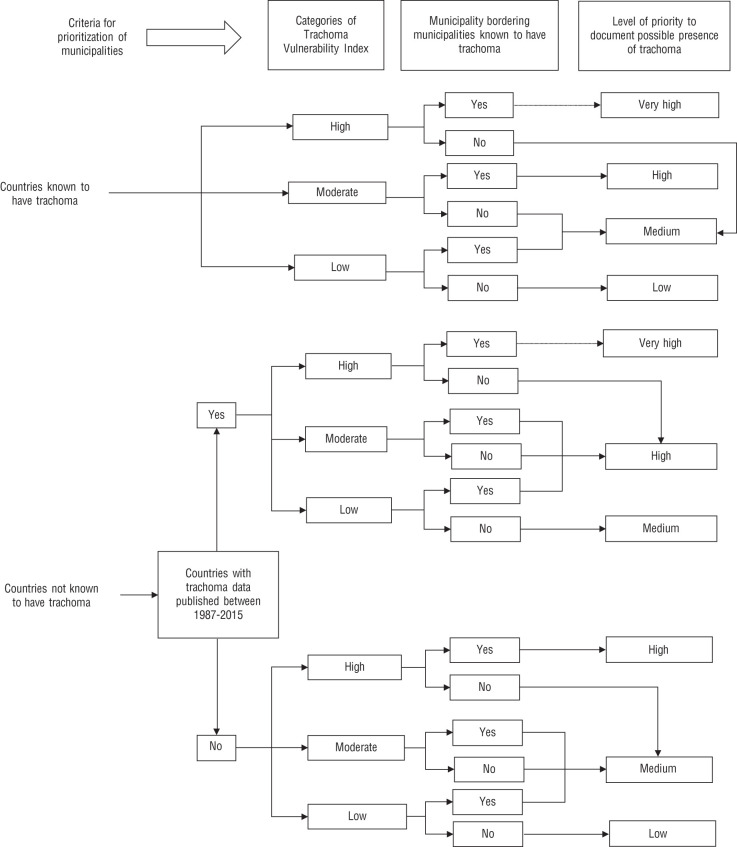
Flow chart of the combination of criteria for the prioritization scale of municipalities requiring surveillance activities for trachoma in Latin America

***Source:*** Prepared by the authors from the study results.

The prioritization scale of municipalities in the countries with trachoma data published in 1987 – 2015 resulted in three levels: very high, high, and medium. For the group of countries without any data published in that period, the prioritization scale resulted in three levels: high, medium, and low. The distribution analysis was done for all municipalities and each priority level. Figure 1 shows the flow chart of the criteria combined for the prioritization scale.

Municipality data for the 22 countries was compiled and organized using Microsoft Excel™ 2010 (Microsoft Corp., Redmond, Washington, United States) and included: (a) the risk factors to calculate the TVI; (b) the history of trachoma in countries not known to have the disease; (c) the municipalities bordering municipalities where trachoma was known; and (d) the prioritization scale of municipalities. Stata® Statistical Software Release 13.1 (StataCorp LP, College Station, Texas, United States) was used for statistical analysis of the municipality prioritization scale. ArcGIS 10.0 (Environmental Systems Research Institute, Redlands, California, United States) was used to draw the map on the prioritization scale. The map was created using the Second Administrative Level Boundaries cartographic digital database (http://www.ungiwg.org/coreDB).

## RESULTS

Each component of the prioritization scale and the classification of the 10 090 municipalities by priority level for trachoma surveillance are described below.

### 

#### Trachoma vulnerability index.

The mean of the TVI for 10 090 municipalities in 22 countries was 15.62% with a standard deviation (SD) of 11.87 and a range of 0.00% – 92.17%. A total of 2 522 municipalities had a high TVI (the 75^th^ percentile or greater, ≥ 22.73%); 2 523 had a moderate TVI (from the 50^th^ to below the 75^th^ percentile, ≥ 13.10% and < 22.73%, respectively); and 5 045 had a low TVI (below the 50^th^ percentile, < 13.10%). In Nicaragua and Peru, more than 50% of municipalities were in the high TVI category.

#### Historical reporting in countries not known to have trachoma.

Published studies were found for seven countries (Argentina, El Salvador, Haiti, Honduras, Paraguay, Peru, and Uruguay). The studies in Argentina, El Salvador, Honduras, Paraguay, Uruguay, and one in Peru were national rapid assessment surveys on preventable causes of blindness in the adult population, conducted in 2012 – 2015 (27 – 32). The study in Haiti was a serological survey in children under 5 years of age conducted in one community in 2012 (33). A study in Peru, published in 2007, reported cases of conjunctivitis in indigenous people 18 – 68 years of age, who were identified at an ophthalmological institute and tested for *C. trachomatis* infection (34). Another study in Peru, published in 2016, gave an extensive review on the history of trachoma from 1895 – 2000 (35) divided into two time periods (1895 – 1960 and 1983 – 2000). For the second period, the authors described the occurrence of trachoma in 10 of the country’s 25 departments.

Suspected history of trachoma was found in only three countries (El Salvador, Haiti, and Peru) where 32 observations were identified geographically (28, 31, 33 – 35). Data from El Salvador not identifiable at the subnational level were assumed to be from all of the country’s 262 municipalities (28). Data from Peru not disaggregated at the subnational level were assumed to correspond to all 61 municipalities in departments reporting cases (31, 34).

#### Municipalities bordering municipalities known to have trachoma.

Within countries known to have trachoma, 4 107 municipalities shared borders with at least one municipality known to have trachoma: Brazil, 3 887 municipalities; Guatemala, 165; and Colombia, 55. In countries not known to have trachoma, 191 municipalities shared borders with at least one municipality known to have trachoma in a country known as endemic: Argentina, 42 municipalities; Bolivia, 28; Ecuador, 27; Guyana, 4; Paraguay, 40; Peru, 14; Suriname, 6; Uruguay, 5; and Venezuela, 25.

#### Municipalities requiring trachoma surveillance activities.

Brazil, Colombia, and Guatemala have 7 010 municipalities, of which 147 were known to have trachoma based on country reports to PAHO/WHO (36). Of the remaining 6 863 municipalities where the trachoma situation is unknown, the prioritization scale analysis indicated that 1 053 municipalities have a very high priority for implementing surveillance because of a high TVI and a border shared with at least one municipality known to have trachoma: Brazil, 973 municipalities; Guatemala, 69; and Colombia, 11. As shown in Table 3, a total of 1 175 municipalities are high priorities for surveillance because they have a moderate TVI and share borders with at least one municipality known to have trachoma (1 079 in Brazil, 73 in Guatemala, and 23 in Colombia).

**TABLE 3.** Municipalities requiring surveillance activities for trachoma in Latin America, by priority level and country, 2017

	Number of municipalities by priority level								
Very high		High		Medium		Low	
Country	n	%	n	%	n	%	n	%	Total
Argentina	0	0.00	0	0.00	81	15.37	446	84.63	527
Belize	0	0.00	0	0.00	204	78.16	57	21.84	261
Bolivia	0	0.00	0	0.00	34	30.36	78	69.64	112
Brazil	973	17.48	1 079	19.39	2 615	46.99	898	16.14	5 565
Chile	0	0.00	0	0.00	1	1.96	50	98.04	51
Colombia	11	0.99	23	2.06	800	71.81	280	25.13	1 114
Costa Rica	0	0.00	0	0.00	0	0.00	81	100.00	81
Dominican Republic	0	0.00	0	0.00	52	33.55	103	66.45	155
Ecuador	0	0.00	12	5.36	131	58.48	81	36.16	224
El Salvador	0	0.00	96	36.64	166	63.36	0	0.00	262
Guatemala	69	20.85	73	22.05	162	48.94	27	8.16	331
Guyana	0	0.00	3	30.00	2	20.00	5	50.00	10
Honduras	0	0.00	0	0.00	141	47.32	157	52.68	298
Jamaica	0	0.00	0	0.00	0	0.00	14	100.00	14
Nicaragua	0	0.00	0	0.00	140	91.50	13	8.50	153
Panama	0	0.00	0	0.00	19	25.00	57	75.00	76
Paraguay	0	0.00	1	0.44	43	18.78	185	80.79	229
Peru	0	0.00	60	30.77	116	59.49	19	9.74	195
Suriname	0	0.00	5	8.06	39	62.90	18	29.03	62
Trinidad and Tobago	0	0.00	0	0.00	0	0.00	15	100.00	15
Uruguay	0	0.00	0	0.00	5	26.32	14	73.68	19
Venezuela	0	0.00	6	1.79	66	19.64	264	78.57	336
TOTAL	1 053	10.44	1 358	13.46	4 817	47.74	2 862	28.36	10 090

***Source:*** Prepared by the authors from the study results.

**FIGURE 2.**
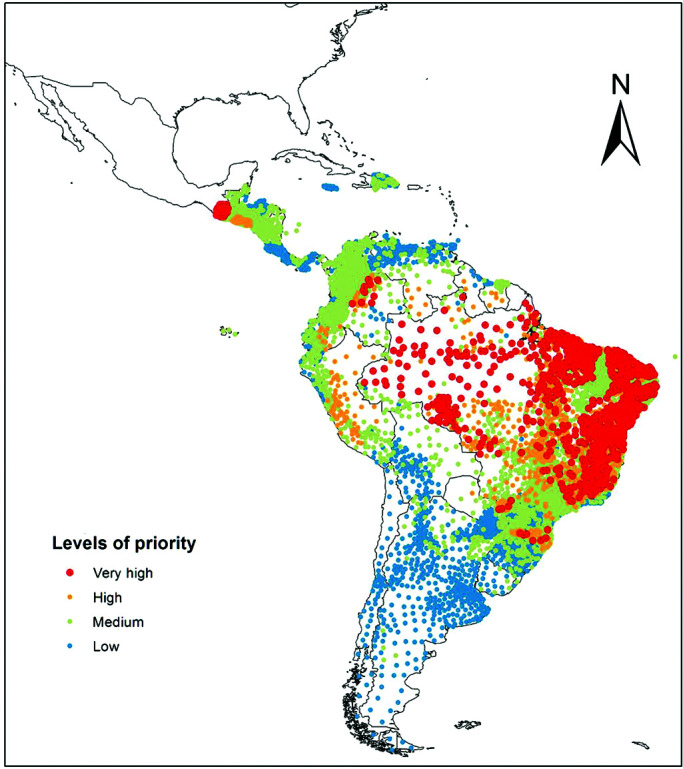
Classification of municipalities for surveillance of trachoma by priority level in 22 countries of Latin America, 2017

***Source:*** Prepared by the authors from the study results.

The prioritization scale indicated that in the 19 countries not known to have trachoma, there are 3 080 municipalities of which 5.94% (183 municipalities) have a high need to implement trachoma surveillance for one of the following reasons: they have published data on the occurrence of trachoma in 1987 – 2015; they have a moderate or low TVI, but share a border with at least one municipality known to have trachoma in another country; or they did not have published trachoma data in 1987 – 2015, but have a high TVI and share a border with at least one municipality known to have trachoma in another country. Of these 183 municipalities, El Salvador had 96; Peru, 60; Ecuador, 12; Venezuela, 6; Suriname, 5; Guyana, 3; and Paraguay, 1. Figure 2 shows the municipalities classified by priority level for trachoma surveillance in the 22 countries under study.

## DISCUSSION

The 1 053 municipalities identified as very high priorities for trachoma surveillance are in Brazil, Colombia, and Guatemala. The population of these municipalities ranged from 1 310 – 161 905 residents. Grouping municipalities to form evaluation units (areas with populations from 100 000 – 250 000 inhabitants) for surveillance activities could be feasible when they are geographically close (5). In these countries, there were 147 districts that warranted interventions for trachoma elimination in 2016 (36). However, mapping has not been completed to confirm that these countries do not have any additional endemic municipality. It has been recommended that Brazil perform a reassessment of its trachoma epidemiological situation (26).

In the other 19 Latin American countries, it is imperative to collect data that can contribute to validating trachoma elimination in the Region of the Americas. Our study’s prioritization scale indicates that there are 183 municipalities in seven countries with a high priority for surveillance activities. Five of these countries have municipalities in the Amazon basin (Ecuador, Guyana, Peru, Suriname, and Venezuela); one has municipalities in the Chaco region (Paraguay); and one is in Central America bordering Guatemala (El Salvador). The absence or prevalence of trachoma in these countries must be documented to determine the extent of the disease in Latin America. In June 2017, in a Peruvian district in the Amazon jungle that borders Brazil and Colombia, such an effort identified another municipality with trachoma. Efforts to implement trachoma surveillance activities should be integrated with other surveillance conducted in the same communities, e.g., soil-transmitted helminth infection, Chagas disease, malaria, and others.

Serology might be useful to surveillance by collecting immunological information by age group in different epidemiological settings. Although the cut-offs are not established for the level of trachoma antibodies that constitutes a public health problem and there remain issues with specificity (37), additional survey data could contribute to characterizing the usefulness of the serological profile for trachoma.

Our study identified nine other countries not known to have trachoma, but where 677 municipalities were designated as having a medium priority for surveillance (Argentina, Belize, Bolivia, Chile, Dominican Republic, Honduras, Nicaragua, Panama, and Uruguay). Trachoma rapid assessment could be a first step in documenting any occurrence of trachoma in these countries (38).

The possible occurrence of trachoma in Haiti needs to be investigated. Although our study excluded Haiti for its lack of data to determine TVI at the municipal level, the country’s social and economic conditions—together with deficient access to basic services and the serological findings from a study of *C. trachomatis* antibodies (33)—make it logical to propose a population-based survey of trachoma. Note that serological findings are not necessarily indicative of trachoma when there is a high rate of exposure to urogenital *C. trachomatis* at sexual debut (37).

### Limitations

The study had some limitations to consider. There was insufficient published information on countries known to have trachoma, particularly Colombia and Guatemala. This shortcoming was partially addressed with reports from the Regional trachoma meetings and country reports to PAHO/WHO. Even less published information was available for areas beyond the three countries known to have trachoma in 2016. The information available came primarily from rapid assessments of causes of blindness (including the identification of TT cases and corneal opacity). These are not necessarily indicative of current trachoma presence because only individuals 50 years of age or older were examined—a group likely to have suffered repeated, active infections as children, but not necessarily in the same geographic area captured by the survey. This might have caused an overestimation of the number of municipalities prioritized for trachoma surveillance. For El Salvador we likely overestimated the municipalities requiring trachoma detection because, based on a national survey reporting corneal opacity caused by trachoma, we classified the entire country as having historical information published in 1987 – 2015. Several of the studies published in 1987 –2015 focus on a series of cases; these are not robust studies on trachoma as a public health problem. This reinforces the view of trachoma as a neglected infectious disease in Latin America. In addition, data from the population and housing censuses of some countries are more than 15 years old, which affects the analysis of risk factors because social and economic conditions may have changed significantly. We also did not exclude urban areas from TVI analysis, which might have overestimated the number of municipalities given priority for trachoma surveillance. Lastly, most of the Caribbean countries lacked data on trachoma-related risk factors at the municipal level and could not be included, which produced an incomplete picture of trachoma in the Region of the Americas.

### Conclusions

The prioritization scale identified 1 053 municipalities in Brazil, Colombia, and Guatemala as *very high* priority for trachoma surveillance, and another 183 municipalities in Ecuador, El Salvador, Guyana, Paraguay, Peru, Suriname, and Venezuela as *high* priority. This study was a first attempt at assessing the need for trachoma surveillance in countries with little data available. This prioritization scale could be useful to many countries in Latin America that need to understand their current trachoma situation, but where the cost and complexity of carrying out baseline surveys would impede or slow their efforts.

We recommend that (a) countries designated as very high and high priority for trachoma surveillance include surveillance activities in their public health agendas to determine the absence or prevalence of trachoma; (b) public health and research groups connect with researchers interested in survey methodologies (i.e., serology as a trachoma surveillance tool, the use of image capture modalities, integrating surveys for several diseases) and predictive statistical models to ensure cost-effective surveys and to provide further evidence of where trachoma continues to be a public health problem; and (c) that the prioritization scale be tested by comparing its results to those of surveillance activities in order to validate its usefulness as a tool for identifying communities at risk of trachoma.

### Author contributions.

MISD and AFB conceived and designed the study. MISD performed the study. MISD and AFB organized and analyzed data. MISD led the writing process. All authors contributed to interpretation of results and edited the paper. All authors reviewed and approved the final version.

### Funding.

The authors received no specific funding for this work. This work was conducted by PAHO/WHO through the biennial working plan of the Regional Program of Neglected Infectious Diseases. The funders had no role in the study design, data collection or analysis, decision to publish, or preparation of the manuscript.

### Disclaimer.

Authors hold sole responsibility for the views expressed in the manuscript, which may not necessarily reflect the opinion or policy of the RPSP/PAJPH and/or PAHO.
